# Practical Surgery. With One Hundred and Twenty Engravings on Wood

**Published:** 1838-04

**Authors:** 


### Art. X.

*Practical Surgery. With one hundred and twenty Engravings on Wood.* By Robert Liston, Surgeon.—*London*, 1837. 8vo. pp. 494.

The object of Mr. Liston's volume is to present to students of surgery and young practitioners, a plain, common-sense view of the most important injuries and diseases which are met with in practice, unencumbered by speculations or theories, and accompanied by simple directions how to conduct the treatment. In noticing the work, it appears to us that we shall render Mr. Liston the most justice, and our readers the most service, by adverting chiefly to those points in which he recommends a different practice to that commonly adopted. For the mere elements of operative surgery, the common points of professional knowledge, we must recommend the perusal of the work itself.

In the first chapter many instructive remarks are made on the division of parts by the knife. The very first observation is important. It conveys useful advice to the junior surgeon, and we wish we could add that it was always remembered or acted upon by the veteran practitioner. When once the necessity for the performance of a surgical operation is clearly indicated, the first consideration that ought to occupy attention is the mental and bodily condition of the patient. If these are in a favorable state, "the less delay in resorting to operative procedure, and the shorter the period of uncertainty and suspense in which the patient is kept, the better." In hospital practice, it too frequently happens, that, when the performance of an operation is determined upon, the patient, perfectly aware of the trial he has to undergo, is left to the next "operating day," that every student may have an opportunity of witnessing the operation. He necessarily broods over the sufferings he has to undergo, and neither his mental powers nor his bodily strength can be in so favorable a state for subsequent recovery as if the surgeon had acted without delay, when the necessity for operating had been established. The following hints, too, may not be useless to the old as well as young surgeon.

"The operator should consider well the place which he himself should occupy during the proceeding, so that he may, without awkwardness or change of position, be able to effect his object efficiently and gracefully; he will also act wisely in general, so to dispose the instruments and apparatus, that he can at once put his own hand upon them, and thus render himself independent of lookers-on, who, in nine cases out of ten, owing to anxiety or curiosity, or to their hurry and agitation, hand anything but what may at the instant be required. If he have had experience in such proceedings, he will previously have ascertained that everything is in order, that the cutting instruments have good points, that their edges are keen, and that the joints of forceps and scissors move freely and readily." (P. 6.)

Upon more than one occasion we have seen confusion arise during the performance of an operation, and one instrument confessedly substituted for another, because the assistants were not aware of the particular implements the operator might prefer, and they were consequently not at hand.

Upon the subject of the "Union of Wounds," Mr. Liston observes that, so long as oozing of blood continues, no purpose can in general be answered by putting the divided surfaces into close contact. If this practice be pursued, the blood which oozes away from the smaller vessels is necessarily prevented from escaping. The consequence must be infiltration of the thin and loose open cellular tissue around, the distention of the cavity of the wound, and the separation of the surfaces and deep-seated parts. The following sketch of routine practice appears to us correctly drawn, and we really think the author is not amenable to the charge of exaggeration.

"A sort of routine practice has been long pursued in dressing wounds. They are put together without delay, and their edges squeezed into apposition and retained so, by various means, such as sutures, plasters, compresses, and bandages. They are carefully covered up and concealed from our view for a certain number of days. Then the envelopes of cotton and flannel, the compress cloths, the pledgets of healing ointment and plasters are taken away, loaded with putrid exhalations and a profusion of bloody, ill-digested, fætid matter. A basin is forthwith held under the injured part, and the exposed and tender surface is deluged with water from a sponge, and then well squeezed and wiped. Then comes a re-application of retentive bandage, of the plaster, of the grease mixed with drying powder, and surmounted by some absorbent stuff, as charpie or tow, to soak up the discharge. This is not unaccompanied with pain, often more complained of than that attendant upon the original injury or operation. This process is repeated day after day, the patient is kept in a state of constant excitement, and often falls a victim to the practice, worn out by suffering, discharge, and hectic fever. It would here serve no purpose to detail the mode of cutting the plasters, and of spreading the pledgets; nor would there be any use in giving an opinion as to whether a mixture of the earth called armenian bole, or the impure oxyde of zinc, the tutty or calamine, was the best ingredient to put into the digestive, drying, healing unguents, or as to whether they should be compounded of one kind of animal fat and vegetable oil, or another; nor would any good come of stating how the soiled bandages and filthy straps are to be removed, whether they are to be cut off or removed first from one end and then from the other. The system is a bad one, the applications filthy and abominable; the whole proceeding outrages nature and common sense. The wound is put in a forcing-bed, as it were; excited action, beyond what is required, is hurried on, and the consequence is, that union seldom, if ever, can or does take place. A suppurating surface, on the contrary, with profuse discharge, and a very tedious cure, if any, is obtained." (Pp. 28-29.)

Surfaces are not disposed to unite for many hours after the division and separation has occurred. So long as oozing continues, there is no good end to be achieved by their close apposition. It is only when reaction has occurred, when the vascular excitement around the solution of continuity has taken place, and the circulation has been roused; when plastic matter begins to be secreted and thrown out, that the process can be expected to commence. The edges of a large wound, as in amputation of the extremities, may be approximated in part, as soon as the bleeding from the principal vessels has been arrested: "but the close apposition, and the application of all the retentive means had better be delayed for six or eight hours at least. In the interval, extreme sensibility of the injured parts may be abated, the oozing moderated, and the chance of secondary bleeding much diminished, by covering the parts with lint dipped in cold water, and frequently renewed." This is the best practice, in Mr. Liston's opinion, in wounds made by the surgeon, and those which are accidental, of the incised kind, that are fresh and bleed freely. Mr. L. has a great antipathy to poultices: for example, in speaking of the management of accidentally incised wounds, he says,

"After hæmorrhage has entirely ceased, applications of an agreeable warmth should be substituted, such as poultice of bread and water, or what is much to be preferred for its simplicity, lint of thick texture, and of sufficient size to cover the wound, soaked in tepid water, and that overlaid with an ample piece of oiled silk, to prevent evaporation. Heat and moisture, by which qualities a poultice produces its soothing and beneficial effects, by which the surface is relaxed, its capillary circulation encouraged, and discharge promoted, are thus amply afforded, without any of the weight, putrefactive fermentation, stench, and filth, which is inseparable even from the best and most scientifically contrived epithems and cataplasms." (P. 29.)

The adhesive plaster in common use is objected to: "it does not retain its hold sufficiently long; it is loosened by discharge, it heats the surface, and often gives rise to erythema." For many years a "better sort of plaster" has been used by Mr. Liston and his colleagues in Edinburgh and London. It is composed of a solution of isinglass in spirit, and may be spread for use, as occasion requires, on slips of oiled silk, or silk glazed on one side only, and on the unglazed side. This plaster is kept ready for use by Messrs. Fisher and Toller, of Conduit street. It is cut into strips of the desired breadth, and the adhesive matter dissolved immediately before it is employed by the application of a hot moist sponge. "It is adherent, keeps its hold to the end of the cure, and is quite unirritating." Being transparent, too, the plaster does not prevent any untoward process that may be going on underneath from being observed, and if any fluid collects, an opening can be snipped for its escape. Under some circumstances, and in some instances, the delay advised in bringing together the surfaces of wounds, and closing them permanently may be departed from with great advantage, as when the entire surface can be brought into close and accurate apposition, so that no clot of blood can by any possibility be interposed.

Under the head of *Injuries of Bones*, Mr. Liston includes Fractures of the cranium, of the pelvis, injuries of the spinal column, and fractures of the upper and lower extremities, disunited fracture, and diseases of bones. Passing over many excellent remarks as to the diagnosis of mere concussion of the brain and compression, we come to a practical opinion of Mr. Liston's on the subject of trephining, in which many surgeons would not coincide. It is laid down as a general rule, to which very few exceptions are admitted, that the only true and justifiable reason for employing the trephine, or sawing away any portion of the skull, is to remove pressure from the brain, which pressure must also actually occasion dangerous symptoms. Mr. Lawrence is reported to state in his lectures* "that, in the present day, we have entirely discarded the doctrine of employing the trephine, or instrument of any sort, in all cases of fracture of the skull; and consider it as a measure that we are not to have recourse to, except where there is a depression of the bone, and *that depression of the bone accompanied with symptoms of pressure on the*

* Med. Gazette, vol. vi. 599.

*brain.*" Desault, in the latter years of his life, had entirely abandoned the operation, so numerous had been the unfavorable results of trephining in the Hôtel Dieu. Mr. Lawrence, from what he has seen at St. Bartholomew's Hospital, is "nearly" inclined to coincide with Desault. He says* that, "of the instances in which I have seen the operation performed in this hospital, the greater number have terminated fatally, so that I can cite to you, as far as the experience of this hospital goes, very few instances in which the life of the patient has been saved by the operation of trephining." Mr. Liston has not adverted to this general opinion upon the subject, and we refer to it, as we think the student should know the doubts that exist upon so important a practical subject. We are very far from doubting the solidity of the conclusions to which Mr. Liston's reflections and experience have led him. On the contrary, it appears to us that the observations he makes in the work before us, as well as in the second part of his "Elements of Surgery," and more especially the cases he relates, go very far to shew that the surgeon may often be justified in using the trephine, even though the symptoms of oppressed brain, or disturbance of its functions, do not exist. In the second part of the "Elements of Surgery," there are some excellent cases detailed which tend still more strongly to shew the occasional propriety of using the trephine when no serious symptoms exist, than those which are given in the work before us. We may briefly mention a case in point which we saw, and which was under the care of Mr. Arnott, at the Middlesex Hospital. The patient, a woman, was admitted with the iron leg of a pot sticking in her skull. The house-surgeon removed it with little difficulty. No serious symptoms arose, and she went on quite well. At the expiration of some days the wound suppurated, and Mr. Arnott removed several splinters of bone of the external table of the cranium. There was one larger portion of the inner table of bone, which was loose, but which could not be removed. Fearing the subsequent occurrence of inflammation of the dura mater from this loosened portion of bone, Mr. Arnott trephined the patient, although no symptoms existed of compression of the brain, and the patient's life was perfectly safe at the time. The portion of bone was thus removed, and the patient speedily recovered, and has remained well for three years. It is not, however, to be supposed that Mr. Liston is an advocate for the use of the trephine so frequently and almost indiscriminately as it was once employed in most injuries of the skull. He expressly states "that it is an operation, at this period, very rarely resorted to or witnessed."

For the management of different fractures of the extremities, we must refer to the work. Upon the subject of disunited fractures, cases so extremely distressing to patients, and generally so perplexing to the surgeon, Mr. Liston's experience cannot but be acceptable.

"Various means have been resorted to with a view to set up a process by which consolidation might be brought about. In recent cases, by changing the position of the limb if necessary, and taking great pains to prevent the least movement of the parts, retaining them in very close and accurate contact by means of splints and bandages, the object may be attained. But it is generally necessary to take means previously to promote a certain degree of excited action. The ends of the bones have

* Med. Gazette, vol. vi. 599.

been moved about, and rubbed against each other. Incisions have been made, escharotics applied, and portions of the bone have been sawn off in many cases, without much benefit accruing. The chance of success depends a great deal upon the relative position of the ends. If they overlap, and are in contact, the case may be looked upon as favorable. The action of the vessels of the membrane of the bone can be roused, and the best means of doing so is by the introduction of a perforator betwixt the two portions, followed by a strong needle, with an eye near its point, by which a coarse seton is passed in withdrawing it. Of course an opening will be made with a bistoury in the soft parts, close to the bone, taking care to avoid vessels, nerves, or other organs of importance, before the introduction of instruments to disturb and lacerate the deep parts. If the bones do not overlap, and they very generally are so placed, it will be advisable to make the attempt to put them in that position; a short and firm limb being more serviceable than one that dangles about, weak and unsupported by the muscles. This practice has succeeded in the humerus, which, by the way, is the bone most frequently the seat of false joint, in the thigh, fore-arm, and leg. I have had several cases in which the treatment by seton has been followed by a perfectly successful result in the fore-arm, upper-arm, and leg; I have also, as might be expected, had one or two failures, and have besides declined interfering in several instances, on account of the unfavorable circumstances attendant upon them. The plan I have pursued has been to pull about the parts a good deal at first, to introduce a larger and larger cord, and to remove the foreign body at the end of a few days, eight or ten, as soon in fact as a considerable degree of excited action had arisen in the bone and periosteum, and before it has begun to decline; the limb is then put up with great care, and every chance of the slightest motion taking place guarded against. The object of passing a seton is assuredly not to promote discharge, which is prejudicial to and often enough, when the result of accident, interferes with the union, and gives rise to the necessity for such operations as that now under consideration." (P. 86.)

The chapter *on Injuries and Diseases of Joints* is rich in very instructive matter and interesting cases. Our own experience has furnished us with abundant opportunities of seeing the truth of Mr. Liston's remarks on the subject of the much too-frequent employment of moxas, issues, perpetual blisters, &c., to the destruction of the patient's health, when patience and rest alone ultimately succeeded in effecting the cure of diseased joints.

"In all injuries and diseases of joints," says Mr. Liston, "in the slow, strumous degenerations, white swellings (a most comprehensive term), as in the most violent form of articular inflammation, perfect quietude and repose of the affected part form the most powerful and essential curative indication: neglect this, all other means are found nugatory, and were as well untried. Nothing but disrepute and disgrace can accrue to the profession and professors if hot irons, moxas, and issues continue to be used, as they often are, inconsiderately enough, to the neglect of more powerful and less appalling means." . . . "The sudden improvement in the health of patients worn down by the disease of a joint can be witnessed at any time, in the cases of morbus coxarius, treated on the principle here laid down, at the North London Hospital. In the first stage of the disease, during the period when there is apparent elongation, and also where ulceration has made progress and the limb is shrunk and shortened, great benefit and relief will be found to follow the adoption of this method; the joint is placed extended, the most favorable position it can occupy, should permanent stiffness arise. A splint, similar to that used by the old surgeons, composed of some soft substance, as tow and albumen, described by Scultetus, and delineated by him even to the eggs on a platter, is applied; we use coarse soft lint soaked in a strong solution of gum acacia; it is laid on in strips over the side and pelvis, from the short ribs down to below the knee, and made to embrace the limb fully. A layer of dry lint is first applied, and then two or three others, soaked in the mucilage, follow; this is covered by a fold or two of coarse calico, and the whole retained by a roller. In cases where the limb has been retained for a long period adducted and bent, and where some little trouble and uneasiness has arisen in placing it in a favorable position, it will be advisable to preserve it so by the use of the thigh-splint, as for fracture, for some twenty or twenty-four hours, until the composition dries, and the splint has adapted itself closely to the parts." (Pp. 130-131.)

In acute inflammatory attacks of the joints, active antiphlogistic means must be adopted, so as at once to make an impression upon and extinguish the action. After free general and local bleeding, "fomentations will relax and keep up a determination to the surface; cold lotions, so often and inconsiderately used, must have the opposite effect. It is a thorough routine system, useful in few cases, and productive of aggravation in many." Mr. Liston doubts the good effects of cold applications in any case after inflammation has commenced, however useful they may be before the action has come on, with the view of preventing and moderating it. In chronic swelling of a joint the great object of the surgeon will be to prevent destruction of the cartilages and bones: he must also endeavour to promote absorption of the fluids effused into the synovial capsule and bursæ, and to bring their secreting membranes into a more healthy condition. In the more favorable cases speedy amendment will follow the fixing the articulation, and the application of uniform and gentle pressure.

"This is effected somewhat after the fashion of Mr. Scott's plastering, which has been employed very extensively, and rather indiscriminately, to all and sundry affections of joints, and to many swellings and pains in other parts. A greater part of Mr. Scott's process—all that part of it intended for effect—may well and safely be dispensed with, such as the bathing with camphorated spirit, the mercurial ointment, and a vast deal of the plastering. It is advisable to give support to the lower part of the limb by a roller, to near the affected joint; the surface should then be covered by lint, spread with some gently stimulating application, soap cerate with camphor answers well; the whole articulation is then to be surrounded and supported by long strips of plaster, crossed in various directions, and drawn with a very moderate degree of tightness, so as to give a feeling of support without occasioning uneasiness: the roller is then carried upwards over this dressing and to some extent above the joint. In order to prevent motion of the affected parts, which would tend to keep up and increase the mischief, leather splints should be applied outside." (P. 133.)

The general health must be carefully attended to. The alkalies, sarsaparilla, iodine, are all useful. Mr. Liston, we think, is a little incautious in his utter exclusion of mercurial alteratives. Even in the more painful and dangerous affections of the joints, where the cartilages are probably absorbed, good effects may arise from judicious treatment. Here the principle of preventing all motion, if well followed out, will be found advantageous. Great relief will often be obtained by establishing a permanent discharge from the *neighbourhood* of the diseased tissues.

"This can be done simply, and effectually, without causing alarm, or exciting much pain, by confining with a piece of lint and diachylon plaster, a small bit of caustic potash on the skin near the diseased joint. After the slough separates, the sore is dressed with any simple ointment, and it is deepened and made to discharge freely, when disposed to heal, by a few hours' application of the antimonial ointment. A seton may be preferred in some situations; certainly discharge can be kept up and derivation obtained from the affected parts thus, fully as well as by actual cauteries, moxas, pea issues, or other farrier-like practices. Great care must be taken in the placing of issues; they should be near, but not upon a joint. Serious results have now and then followed their careless application. Diseased action has been increased, the cautery having reached, or even penetrated, the synovial capsule. I write after some experience, and from cases and specimens now before me." (P. 135.)

Under the head of *Injuries and Diseases of Blood-vessels,* Mr. Liston observes that the ligature of the common carotid, has, thanks to Sir Astley Cooper, been placed among the regular operations. Before undertaking any operation for aneurism, more especially by ligature of a vessel near the heart, it will be advisable to see that the circulation is in a quiet state; and, if not contra-indicated, it may be found a safe precaution to diminish the quantity of circulating fluid by one or more venesections beforehand, and a low diet. The effects of disturbing the flow of blood to the brain was much dreaded in earlier times. The disease was looked upon as irremediable, and the patient was left to his fate. "A case is given by J. Bell, in which, so late as 1807 or 1808, a poor woman was allowed to perish by hemorrhage from the sac, under the eyes of himself and colleagues." Both carotids have been tied in the human subject, with but a short period of time intervening, and without any bad effects arising. Mr. Liston objects to the operation of tying the carotid artery, in order to arrest the growth of tumours of the face and jaws; or as a preliminary step to the removal of such diseases.

"The suffering of the patient is thus much prolonged, without his safety being at all enhanced, or the dissection of the tumour in any way facilitated. The flow of blood is quite as effectually commanded by pressure with the fingers on the common carotid of the affected side; pressure on both at the same time has given rise to most alarming convulsive movements in some cases, after great loss of blood had occurred. Even pressure on one trunk is not demanded in many of the operations in question. I believe I have had fully as much experience in the management of tumours of the mouth and jaws, and of the face and neck as any surgeon in this country, and have never seen occasion to tie the carotid previous to or during the operations for their removal; and have never regretted omitting this supposed precautionary measure." (P. 164.)

The arteries running to the thyroid body have been tied, with the view of diminishing the hypertrophy by which it is sometimes effected; "but no good has been answered by such proceedings." Aneurisms are met with at the root of the neck, and so situated that there is no possibility of reaching a sound portion of vessel betwixt them and the heart, so as to interrupt the flow of blood. It has been proposed to treat aneurisms of these vessels close to their origin by ligature on their distal side.

"This is a proceeding," says Mr. Liston, "which, in a favorable case of the kind and at the urgent solicitation of the patient, a surgeon might be induced to adopt, as the only though desperate remedy for an otherwise incurable disease; it is an operation, however, which he would not be warranted in urging a patient to submit to." (P. 172.)

It may be necessary to tie the brachial artery in consequence of wound of the palm, involving the deep or superficial palmar arches, or on account of spontaneous aneurism in this part; a most uncommon circumstance. The following case came under the notice of Mr. Grainger and Mr. Pilcher.

"'A working goldsmith, about forty years of age, of a gouty diathesis, had a tumour formed beneath the ball of the right thumb; it was mistaken for an abscess; on careful examination, I discovered it to be an aneurism, and believed it to be situated between the adductor pollicis and abductor indicis, and to be a disease of the radial artery at its terminal division, probably induced by the repeated, thought slight, blows from the handle of the hammer, which his occupation constantly obliged him to use. I proposed to tie the radial and ulnar arteries immediately above the wrist, provided the ligature around the radial was ineffective in diminishing the tumour and arresting its pulsation. My expectations were realized; the closure of the radial artery was attended with diminution in the size and pulsation of the tumour, but still both remained to rather less than half the previous degree. I immediately tied the ulnar, when the tumour was much reduced in size, and the pulsation completely: slight secondary hæmorrhage occurred from one of the arteries at the seat of the ligature two or three days after the operation, which was arrested by cold water; the case progressed without any further untoward symptom, and was attended with a perfect cure.'" (P. 177.)

In this instance success followed the operation; but Mr. Liston says there was a risk of the tumour being filled with fluid blood through the interosseous, and states that, if such a case were presented to him, he would tie the humoral artery low in the arm. The following remarks are too important to be condensed:

"Recent hæmorrhage from the palm must be commanded by ligature on the divided ends of the vessels, exposed farther, if need be, by dilatation of the wound. If the bleeding has been at first commanded by pressure, as it may be permanently, when but small twigs are implicated, and blood bursts out impetuously again and again, after the tissues have been altered, by inflammatory swelling, abscess, and infiltration of blood, then should the clearing out of the wound, and methodical pressure from the bottom of the cavity not prove effectual, recourse should at once be had to the operation on the brachial. There is no use in trying to include the vessels, even could they be got at readily and safely in the palm; they will not hold a ligature when in this state for any time, nor can ligature on the radial and ulnar be relied upon. Pressure on the vessels is sometimes resorted to in such cases, and in such a way as to operate upon their course, part of the circumference of the limb being uncompressed. Ring tourniquets, as they have been called, are invented and sometimes employed for the purpose; the veins cannot entirely escape obstruction, and the effect of this upon the diseased parts must be apparent. It is a remnant of the old and barbarous surgery. The practice may be successful at a time, and by chance, but if generally employed it must lead to mischief in the deep parts, bones, and joints, and dependence cannot be placed upon it for a cure. The ligature of the vessel under very unfavorable circumstances, might, after all, be called for, or even the amputation of the member." (Pp. 178-179.)

In the next chapter, *on Injuries and Diseases of the Integument and Cellular Tissue*, we again find Mr. Liston strongly deprecating the application of poultices.

"A poultice, always a filthy and uncomfortable application from its weight and stench, may be used; what is much better, equally efficacious, and not liable to objection in any way, is a double piece of lint soaked in hot water, of an agreeable temperature, applied to the part; it is covered by an ample piece of oiled silk, to prevent evaporation, and this dressing, simple enough, but answering every purpose, is removed frequently; the lint may be moistened by merely removing the oiled silk, if the parts are very tender, and if it be not soiled by discharges; all the soothing effect of a poultice is thus produced, without any discomfort. This warm-water dressing is light and nice, and it is changed often if there be any unpleasant odour exhaled; it may be medicated with extract of poppies, or salts of opium, or it may be coloured, or have some aromatic added, if the patient does not put faith in simple means. A great deal has been said about water-dressing, and the merit of introducing it; water has been applied to sores from time immemorial. The simple element, water, was supposed to be congenial to wounds and sores; it was used to cool parts. The water-dressing has been used in my hospital and private practice for a long series of years, as a substitute for poultice, as a means of conveying and preserving heat and moisture, on a surface that should secrete pus for its protection, and as an accompaniment of the process of healing by the second intention." (Pp. 197-198.)

We are by no means disposed to "gulp at every change," as Burton says, but there appears to us much good sense in this proposed innovation upon the old poultice system, and we cannot but wonder that Mr. Liston has not hit one more blow against the "abomination," by asking, how many hours out of the twenty-four does a poulticed patient enjoy the benefit of a *warm* application?

The next chapters treat of *the Restoration of Lost Parts*, and of *Morbid Growths and Enlargements.* We can only touch upon one point discussed here. It was customary to remove the chronic enlargements of the tonsils and uvula by ligature, and, strange to say, this method is still preferred and practised by some surgeons.

"It is a most difficult, tedious, painful, and unsatisfactory proceeding, so far as I have seen and can understand; there is no excuse for persisting in it, no risk whatever of bleeding is incurred, if the incision is practised properly. In many cases, by getting rid of the cause, or waiting until that disappears, until wisdom teeth make their way through the gum, (this may sometimes be accelerated) by the use of strong astringent applications and constitutional treatment, the necessity for any operation may be dispensed with. But in cases where other means have failed, where much inconvenience is felt,—above all, when the isthmus faucium is much narrowed, and any alarming difficulty of respiration has occurred, then the removal should not be delayed. It is by no means necessary to remove the whole tonsil, and the attempt would be attended with the greatest danger." (P. 251.)

We particularly recommend to the notice of the young surgeon the tenth chapter, *on Amputations.* The practical directions that are given in the text are rendered extremely clear and intelligible by numerous well planned and executed sketches.

In the next chapter, after pointing out the management of wounds and foreign bodies in the eye, *obstructions* in the nasal duct, and foreign bodies in the pharynx, Mr. Liston comes to the subject of *transverse Wounds of the Larynx and Trachea*, and gives a very good case that well exemplifies the danger of the too-common practice of bringing wounds of the throat together by sutures, bandages, &c., before the oozing of blood has completely ceased, and "the surface is glazed."

"Great and imminent danger, as has already been pointed out, arises from the closure of the wound and the consequent inhalation of blood, but even though the air-tube is not opened, the patient may be put in great jeopardy by the close apposition of the edges of the incision. The blood is apt to accumulate in the cavity, and coagulates; hæmorrhage is thus kept up; the size and pressure of the clot may even interfere with the function of respiration. A young woman was admitted into the North London Hospital, some months ago, on account of a transverse wound of the fore part of the neck over the upper part of the thyroid cartilage. It was ragged, had been inflicted by repeated application of the cutting instrument, and the integument had been somewhat detached from the subjacent parts. The wound was stitched closely before her admission; the then house-surgeon, disregarding the common-sense view of the case, and despite of the principles which I had over and over again inculcated, very foolishly did not throw the edges loose, even though the wound had bled repeatedly and the patient did not breathe with freedom; I was making my visit in an adjoining ward, when the nurse rushed in to say that her patient was dying of suffocation, and she was correct in her statement; the patient was gasping for breath, with a livid countenance, and scarcely any pulse. The stitches were immediately cut out and a large clot removed. There was no further bleeding, the breathing became unembarrassed, and all did well." (P. 339.)

In wounds which do not penetrate very deeply, the patient can take sufficient support at any stage of the case; but, when the pharynx is implicated, it will be necessary, from the first, to convey, at proper intervals, liquid nourishment into the canal beyond the wound. "There is no use in passing long tubes into the stomach for this purpose; a large elastic catheter and gum bottle will be found quite sufficient for the purpose."

"A great error is sometimes committed in the treatment of cases of cut-throat. The patient is fed through the wound in the neck, the contraction is not favoured by position, and the surfaces are permitted to cicatrize separately; the voice is consequently lost, the patient is rendered perfectly incapable of exertion, not having any control over his respiration, and being thus unable to keep his chest expanded. The patient is, moreover, put in great jeopardy; he is subject to bronchitic attacks and to inflammatory œdema of the orifice through which the air enters; he may thus be cut off suddenly, if in the hands of ill-informed or inexperienced persons, or he may be worn out by cough and profuse expectoration. It is possible occasionally, to remedy even such mismanaged cases; the contracted air-passage above may be widened by the introduction of instruments, and the edges of the wound pared and brought together. A very remarkable case of the kind, which occurred in my practice, is detailed in the 'Edinburgh Medical and Surgical Journal,' vol. xciv. p. 118, and in the 'Elements of Surgery,' vol. ii. p. 243. The patient had the opening through which she had breathed entirely for many months, and in which, in fact, she wore a large round tracheotomy tube, closed, after dilatation of the opening leading upwards into the glottis, which was apparently almost obliterated; and although it was necessary to perform tracheotomy on account of the swelling which supervened and threatened suffocation, a few hours after the removal of a piece of elastic tube which had been worn for many days in the trachea, a perfect recovery took place, the breathing became free, and the voice was almost perfectly restored." (P. 341.)

After taking a general view of the signs and treatment of foreign bodies in the windpipe, Mr. Liston lays it down as his opinion that there can be no question when a foreign body has entered the air-tube; that the sooner it is got rid of the better, whether it lodges in the larynx, floats loosely in the trachea, or is impacted in one of the bronchi. In such cases the practitioner may easily be thrown off his guard by the long intervals of cessation from any serious or even troublesome symptoms; but the patient, so long as any foreign body is in either of these situations, must be in danger from the occasional and very violent fits of coughing to which he will be subject. If he escape from this hazard, he has to encounter the great risk of the supervention of organic disease of the lungs, which may not, it is true, take place for a very long period; but the records of surgery teach us that it will very generally occur unless the foreign body is removed by the efforts of nature or the assistance of art.

Mr. Liston relates Mr. Duncan's case, which we believe is the only one on record in which a foreign body was discovered in, and successfully removed from, the bronchus by operation.* Other cases are adverted to, in which it may become necessary to resort to the operation of tracheotomy, on account of obstruction to the free entrance of air into the lungs, caused by disease at the top of the tube. It is more frequently demanded in cases of œdema of the glottis than from any other cause; "and it is generally attended with a happy result." Mr. L. states that diseases of the larynx are extremely common in the northern metropolis; and that he must have performed the operation nearly twenty times, and with almost uniform success, for chronic swelling, with urgent and threatening symptoms. He objects to the removal of an oval portion of the

* Lancet, vol. ii. 1833-34, p. 419.

tube, for the purpose of keeping the wound patent: he regards this expedient as difficult to accomplish; believes that it does not serve the purpose long, and that it is apt to be followed, after the healing of the wound, by inconvenient and dangerous narrowing of the trachea. He prefers the introduction of a tube, and says "that, after a few minutes, all irritation from its presence ceases." We doubt this speedy cessation of irritation from the presence of the tube as a general fact, and we think that the objections stated by Mr. Liston to the removal of a portion of the trachea more imaginary than real. The practice is sanctioned by Lawrence, White, Carmichael, and Sir C. Bell; and it has been adopted by Mr. Arnott, at the Middlesex Hospital, with safety and success.

The section on *Injuries and Diseases of the Rectum and Anus* is very interesting; but, as we shall have occasion to notice these subjects in another article, we can merely advert to them here. Mr. Liston ridicules the attempts that have been made to mystify the subject of diseases of these parts, and to separate them in a great measure from general surgery: he contends (and justly too, in our opinion,) that there is no such difficulty as has been supposed in understanding their nature.

Mr. Liston commences the subject of *Injuries and Diseases of the Genito-urinary Organs* with a remark to which we would willingly object, but, in truth, we cannot. Our own experience has impressed the mortifying fact upon our minds " that by far the greater number of grave and serious injuries of the urethra and bladder have been inflicted by pretenders to surgical knowledge; and that a considerable number of diseases which come under the notice of surgeons are the product of mismanagement on some previous occasion." In the use of bougies, care and gentleness are particularly insisted upon; and Mr. Liston prefers a small silver catheter, if there is reason to suppose the contraction is considerable: if not, a plated metal bougie of moderate size, slightly curved throughout, and smeared with some bland liniment, may be used. These, he assures us, are much superior in every respect to the plaster, gum, or other soft and pliable bougies. There is no possibility of guiding the points of the latter, or of ascertaining what direction they take.

> " Retention, when the prostate is enlarged, can in general be readily relieved if a proper instrument be used: in many such cases, the bladder cannot possibly be reached with those of ordinary length. The prostate catheter should be made of silver, and at least three inches longer than those employed for other purposes; the beak should be long, and the curve considerably greater. The careful employment of such an instrument will generally be followed by a successful result; whereas, attempts with short and elastic catheters must almost certainly end in disappointment to the practitioner and great injury to the patient. Innumerable cases have been presented to me, in which, for days, persevering attempts have been made to relieve an over-distended bladder: nothing but blood, and that in abundance, has flowed. It has then been imagined that the bladder was full of blood, and means have been employed, such as exhausting syringes and injections of warm water, to break down and extract the coagula supposed to exist. A long catheter has been at last used, with the effect of freeing the bladder of many pounds of high-coloured urine, but nothing else." (P. 389.)

In Mr. Liston's opinion, the operation of puncturing the bladder has been, and still is, much more frequently performed than there is any occasion for. It was not once performed during a series of years, when he was assistant-surgeon and surgeon to the Royal Infirmary of Edinburgh; and it has been performed but once, and that before he joined it, at the North London Hospital, since it was opened; although many cases of bad urinary disease had been admitted at both institutions.

Mr. L. is an advocate for the operation of lithotrity, as it is at present improved and simplified. He regards it as now applicable to a vast variety of cases, and thinks it likely to supersede, in a great measure, the cutting operation for stone. We refer to the work for an account of the cases which are adapted to this practice, and for the mode of employing it.

"The operation of lithotomy, if circumstances are favorable, if the patient's health is tolerably good and his kidneys sound, is very satisfactory in its results when properly performed with few and simple instruments, and on a good plan. The incision of the perineum, and of the prostate to a limited extent, is to be preferred in almost every case. The operation on the gripe, and the recto-vesical method, are not now in the list of regular and established operations. The operation above the pubes, still practised occasionally, is not to be resorted to from choice. If a stone of such a size as could not pass readily through the outlet of the pelvis, perhaps contracted unnaturally, were ever met with, the high incision might be thought preferable." (P. 408.)

As Mr. Liston has performed the lateral operation of lithotomy nearly a hundred times, his practical directions upon this important subject deserve the maturest consideration, and we will not weaken them by an abridgment.

In the chapter on *Injuries and Diseases of Serous Cavities*, Mr. L. lays it down as a rule, "that the evacuation of purulent fluid from the cavity of the pleura must be had recourse to as soon as its existence is ascertained." He has known instances, and we have known similar ones, in which the operation has injudiciously been delayed until the matter was about to make its way to the surface, by the absorption of the intercostal muscles. "Pointing and fluctuation must not be waited for here, as in many other situations." Much difference of opinion once existed as to the time when the operation of empyema should be had recourse to, but we believe that, in the present day, the great majority of surgeons will support the general rule laid down by Mr. Liston. The following account of a distressing class of cases is important:

"Watery swellings of the scrotum are also met with; a result of injury or of some disease in the neighbourhood. In inflammatory œdema of the part, the serosity occupies the cellular tissue. I have seen a number of these cases following sores of the prepuce or glands, sores or fistulæ about the anus; occasionally, also, this swelling, with erythema of the scrotum, arises without any local cause that can be discovered; sometimes it has been attributed to slight injury. In hospital practice, several cases of the kind have now and then presented about the same period. This distension of the scrotum is sudden and considerable; the tumour is slightly red and shining, and very soon a dark or greyish patch is perceptible at the lower part. Unless free incisions are resorted to, the part suspended, and active general and local means employed, the cellular tissue, the skin, and the coverings of the testis, will be lost as effectually as if urine had been extravasated. I had no less than six such cases in the Edinburgh Hospital at one time, in an unhealthy season, and in different stages of their progress. The loss of cellular tissue will depend upon the period at which the case is presented, and the activity and judgment with which the treatment is conducted." (P. 429.)

The work concludes with a very instructive chapter on *Hernia*, and a brief notice of the surgical treatment of *Congenital Deficiencies and Deformities*.

The impression made upon us by the attentive perusal of Mr. Liston's work is extremely favorable. As far as we can judge, there is no part of it which the young surgeon can study without great advantage; and there are many original observations interspersed in it, which merit the attention and consideration of experienced practitioners. If the work had been published anonymously, no doubt would have been entertained but that it came from the pen of one who was well fitted for such a task by extensive experience and sound judgment.

---

